# Specific leaf area modulates but does not explain the association between abiotic stress tolerance and insect feeding guild prevalence in Northern Hemisphere woody plants

**DOI:** 10.1093/aob/mcag065

**Published:** 2026-03-19

**Authors:** Andrea Cerdeira-Pérez, Lauri Laanisto, Nicola Pavanetto, Giacomo Puglielli

**Affiliations:** Departamento de Biología Vegetal y Ecología, Facultad de Biología, Universidad de Sevilla (US), Av. de la Reina Mercedes, 6, Sevilla 41012, Spain; Chair of Biodiversity and Nature Tourism, Institute of Agricultural and Environmental Sciences, Estonian University of Life Sciences, Kreutzwaldi 5, Tartu 51006, Estonia; Chair of Biodiversity and Nature Tourism, Institute of Agricultural and Environmental Sciences, Estonian University of Life Sciences, Kreutzwaldi 5, Tartu 51006, Estonia; Department of Life Sciences, University of Trieste, Licio Giorgeri 10, Trieste 34127, Italy

**Keywords:** Abiotic stress, insect herbivory, feeding guilds, plant functional types, woody plants

## Abstract

**Background and Aims:**

Plant adaptations to abiotic stress and insect herbivory may share functional bases, yet large-scale evidence linking stress tolerance strategies to herbivory patterns remains limited. We tested whether abiotic stress tolerance is associated with insect herbivory patterns in Northern Hemisphere woody plants, and whether specific leaf area (SLA) and plant functional type (PFT) modulate this relationship.

**Methods:**

We combined data on 5927 lepidopteran and hymenopteran species with 645 woody plant species from three PFTs (deciduous angiosperms, evergreen angiosperms, evergreen gymnosperms) for which information on their shade, drought, cold and waterlogging tolerance was also available. We then modelled the prevalence of three feeding guilds (chewers, borers, miners) as a function of stress tolerance, SLA, and their interaction at both species and assemblage levels.

**Key Results:**

Assemblage-level associations were substantially stronger than species-level patterns (54 % vs. 18 % of significant effects, respectively). Despite significant SLA × stress tolerance interactions, these did not provide a clear mechanistic explanation of the observed patterns. In addition, spatial autocorrelation in model residuals was substantial (mean Moran’s *I* = 0.66), and most of the observed effects were not robust to correction for spatial dependency.

**Conclusions:**

Abiotic stress tolerance and SLA interact to shape herbivore guild composition, but SLA modulates rather than mediates this relationship, defining a context within which other, unidentified factors (e.g. most specific defence traits) operate. The strong spatial structure in herbivore communities probably reflects biogeographical processes (dispersal limitation, host specificity, regional species pool assembly) that operate independently of contemporary trait distributions. Understanding plant–herbivore interactions at macroecological scales requires integrating trait-based filtering with historical and biogeographical constraints.

## INTRODUCTION

Plants are continually exposed to abiotic and biotic stressors that shape their growth, survival and evolution (Marquis, 1992; Stam *et al*., 2014). Among biotic stressors, insect herbivory exerts pervasive selective pressures by altering plant phenotype, fitness and population dynamics (Barton and Hanley, 2013; Agrawal and Maron, 2022). Insects exploit plants through combinations of diverse feeding niches (leaves, stems, reproductive organs) and modes (chewing, mining, boring) forming ‘feeding guilds’ that group species by function rather than by taxonomy (Powell *et al*., 1998; Boivin *et al*., 2019). These guilds capture how herbivory impacts different plant organs and offer a framework to link feeding strategies with broad plant adaptive syndromes and morpho-functional traits (Haavik and Stephen, 2023).

Plant adaptations to biotic and abiotic stress are deemed interdependent, because traits conferring tolerance to resource limitation or physical stress may also deter herbivores (Agrawal and Fishbein, 2006). Testing for this association requires combining extensive information on plant adaptive syndromes in relation to abiotic stresses and associated morpho-functional characteristics. Key abiotic stressors, such as drought, cold, shade and waterlogging, vary strongly in forest ecosystems worldwide, constraining the diversity of tolerance strategies across species (Niinemets and Valladares, 2006; Niinemets, 2010; Puglielli *et al*., 2021). In woody plants of the Northern Hemisphere, these strategies can be summarized within a stress tolerance space (STS) defined by two orthogonal axes: a drought–cold/waterlogging trade-off, and an independent shade tolerance gradient (Puglielli *et al*., 2021; Pavanetto *et al*., 2024*a*, *b*). The STS provides a framework for quantifying species’ abiotic stress tolerance syndromes and exploring how these syndromes intersect with other ecological dimensions (Puglielli *et al*., 2022), including mapping the distribution of different feeding guilds along broad stress tolerance gradients.

From a morpho-functional perspective, specific leaf area (SLA), a central axis of the leaf economics spectrum (Wright *et al*., 2004), is a key functional trait related to abiotic stress tolerance. Recent work mapping SLA variation within the STS (Pavanetto *et al*., 2024*a*) has shown that this trait in fact captures a key axis of differentiation in tolerance strategies: drought-tolerant plants are generally associated with lower SLA and leaf nitrogen content (on a mass basis), but higher specific stem density and seed mass compared to cold–waterlogging-tolerant plants; a similar pattern was found when comparing shade-tolerant and shade-intolerant plants. Thus, variation in SLA reflects coordinated changes in functional strategies along the stress tolerance axes defined by the STS. SLA variation may also shape the distribution of feeding guilds along the STS axes. Low-SLA species are characterized by tough, structurally defended leaves that deter external chewers through physical toughness but may provide sufficient tissue volume and protected microhabitats for endophytic miners or favour insect herbivore guilds that do not rely on leaf tissues, such as borers (Peeters, 2002; Peeters *et al*., 2007; Sinclair and Hughes, 2010; Tooker and Giron, 2020). The opposite pattern might be expected for high-SLA species producing thin, nutrient-rich leaves that are more palatable for chewing insects. Such shared functional bases suggest that abiotic stress tolerance strategies may co-vary with herbivory patterns (Züst and Agrawal, 2017) in an SLA-dependent fashion. However, large-scale evidence for these associations remains limited (Koricheva *et al*., 1998; Koricheva, 2002; Garcia *et al*., 2021).

Importantly, SLA variation is further nuanced by plant functional type (PFT), defined here by combinations of leaf habit (deciduous or evergreen) and taxonomic group (angiosperm or gymnosperm). PFTs occupy distinct regions of the leaf economics spectrum: deciduous angiosperms are characterized by relatively high SLA and short leaf lifespans, evergreen angiosperms span a wide range of SLA values, from thin-leaved in more mesic species to thick-leaved sclerophylls, while evergreen gymnosperms are constrained to consistently low SLA values (Poorter *et al*., 2009). This variation in leaf economics among PFTs suggests that the interaction between SLA and abiotic stress tolerance on insect herbivory patterns may also be PFT-dependent, with the magnitude of SLA effects potentially scaling with the breadth of SLA variation within each PFT.

Here, we investigate how insect herbivory relates to abiotic stress tolerance strategies in woody plants across the Northern Hemisphere. Specifically, we ask: (1) Are distinct abiotic stress tolerance strategies associated with different insect feeding guilds? (2) Does this association depend on SLA and the considered PFT? We combined an extensive dataset on the feeding ecology of 5927 lepidopteran and hymenopteran larvae (Cerdeira-Pérez *et al*., 2025), which together constitute >30 % of the known phytophagous insects (Bernays, 1998), with SLA data for 645 woody plant species defining the STS. The choice of Lepidoptera is further justified by this group representing the greatest diversification and radiation of any group of herbivores on the planet (Brown, 2018), while hymenopteran species were included as the sole feeders associated with certain woody plants under study. By quantifying how the prevalence of different insect feeding guilds varies along the STS axes by PFT while accounting for interspecific differences in SLA, we provide the first large-scale assessment of how realized abiotic stress tolerance strategies are associated with different insect feeding guilds in woody plants. Finally, by integrating a spatially explicit version of the STS (Pavanetto *et al*., 2024*b*) into our analysis, we drew patterns at the species and at the assemblage levels.

## METHODS

### Abiotic stress tolerance data

Plants’ abiotic stress tolerance data were obtained from the STS dataset (Puglielli *et al*., 2021). The STS was defined using data on species-specific estimates of tolerance to shade, drought, cold and waterlogging from datasets of Niinemets and Valladares (2006) and Laanisto and Niinemets (2015), including 799 North American, European and East Asian woody shrubs and trees. In these datasets, species’ abiotic stress tolerance data were homogenized according to a uniform five-level scale continuously varying between 1 (very sensitive) and 5 (very tolerant). The formalization of the STS model revealed that two independent dimensions (principal components) capture 72 % of the total variance in species-specific combinations of shade, drought, cold and waterlogging tolerance. The first axis is positively correlated with drought tolerance and negatively correlated with both waterlogging and cold tolerance, defining a waterlogging/cold–drought tolerance trade-off axis: more positive values along this axis indicate increased drought tolerance. The second axis represents a shade tolerance spectrum that adds an independent tolerance dimension to the waterlogging/cold–drought trade-off. More positive values along the shade tolerance spectrum indicate increased shade tolerance. Each pair of coordinates in the STS corresponds to a species-specific combination of tolerances to the four stressors, thereby summarizing species-specific abiotic stress tolerance strategies. The species nomenclature for the STS subset follows the World Flora Online (WFO, https://www.worldfloraonline.org/).

### Plant-associated insect functional guilds data

Insect data were obtained from the InsectGUILD dataset (Cerdeira-Pérez *et al*., 2025). This dataset compiles information on the feeding ecology of 5927 species of phytophagous butterflies, moths (Insecta: Lepidoptera) and sawflies (Insecta: Hymenoptera) known to feed, at their larval stages, on the woody plants that define the STS (we were able to retrieve insect-related data on 79 % of the 799 species defining the STS; see previous section). Within InsectGUILD, the feeding behaviour of each insect species is classified into 14 functional groups or feeding guilds. Feeding guilds are defined as a group of insects exploiting the same plant resources in the same way (Simberloff and Dayan, 1991), based on combining the information on the plant part consumed by the larvae (i.e. the feeding niche) with information on how these are consumed, that is either internally (endophytic mode) or externally (exophytic mode) on the plant.

InsectGUILD also provides a fuzzy clustering-based classification system of the insect species in the dataset (for details see Cerdeira-Pérez *et al*., 2025), where the assignment of species to every cluster is quantified by a degree of membership to a given cluster (continuously varying between 0, complete exclusion, to 1, perfect assignment). Three groups of insects were identified: (1) one cluster (*n* = 3581, 72 % of the total) was dominated by external chewers, mainly leaf chewers (hereafter, chewers); (2) a second cluster (*n* = 436, 9 % of the total) was characterized by internal feeders within reproductive structures and shoots (hereafter, borers); and a third cluster (*n* = 910 species, 18 % of the total) was dominated by internal larvae feeding within leaves or transport tissues (hereafter, miners) (see Table 1 for definitions on feeding groups).

**Table 1.** Insect feeding guild definitions used in this study.

Insect feeding guild	Category and explanation
Chewers (*n* = 3581)	Species that live and feed externally on plant tissues. This functional group was mainly represented by leaf feeders (i.e. species feeding externally on leaf tissues) and a smaller proportion of flower feeders (i.e. insects feeding externally on flowers, flower buds or developing seeds).
Borers (*n* = 436)	Species that bore into and feed within internal plant tissues. This group is mainly represented by fruit borers (i.e. species that bore into and feed on reproductive-structure tissues and/or seeds of angiosperms or gymnosperms) and shoot borers (i.e. species that bore into and feed within the shoots, defining this plant tissue as any young, tender, succulent, current-year, aerial outgrowth from a plant).
Miners (*n* = 910)	Species that live and feed within photosynthetic tissues or transport tissues. This group was mainly represented by leaf miners (i.e. species that excavate galleries or ‘mines’ inside the blade of a leaf or needle, between the epidermal layers) and bark borers (i.e. species that bore into the woody portions of plants, feeding within the living tissue of the stem, trunk, branches or roots, disrupting transport).

*n* indicates the number of insect species belonging to each feeding guild.

This classification encapsulates the main functional trade-offs in insect feeding strategies and encompasses the detrimental effects of insect herbivory on the main host plant functions, namely reproduction, photosynthetic activity, and/or vascular/structural tissues linked to water and sap transport (Cerdeira-Pérez *et al*., 2025). We used the cluster membership values in all the subsequent analyses. Since most of the plant species were associated with multiple insect species, we averaged cluster scores available for each of the three clusters (hereafter, insect feeding groups) at the plant species level (hereafter, species-level dataset). We also retrieved the identity of the insects that make up the assemblages associated with the woody plants to assess the distribution of insect species and genera across PFTs and continental origins.

### Assemblage-level plant and insect feeding guilds data

A recently published dataset (Pavanetto *et al*., 2024*b*) provides spatial information for the plant species defining the STS. Pavanetto *et al*. (2024*b*) aggregated carefully cleaned plant species occurrences from the Global Biodiversity Information Facility (GBIF, https://www.gbif.org/) into equal-area hexagons of 7500 km^2^. We used this hexagon-level dataset for the plant species defining the STS to match plant species occurrences with their corresponding insect feeding guilds information. Since Pavanetto *et al*. (2024*b*) also provided estimates of the prevailing abiotic stress tolerance strategy, as defined by the STS framework, within each hexagon obtained by accounting for the relative (occurrence-based) abundance of plant species in each hexagon, we used the same approach to aggregate insect feeding guilds information at the hexagon level. Thus, our dataset included hexagon-level information on abiotic stress tolerance strategies and insect feeding guilds. Hereafter, we refer to this dataset as assemblage-level data, which included 2791, 1919 and 2245 hexagons for deciduous angiosperms, evergreen angiosperms and evergreen gymnosperms, covering 21, 14.4 and 16.9 % of the Northern Hemisphere, respectively.

### Specific leaf area data

We collated SLA (mm^2^ mg^−1^) data at the species and the assemblage levels from Pavanetto *et al*. (2024*a*, *b*). Pavanetto *et al*. (2024*a*) reported SLA values for species in the STS, which were obtained from Carmona *et al*. (2021) who compiled trait data from public datasets in the TRY Plant Trait Database (v.5.0, https://www.try-db.org/TryWeb/Prop2.php, Kattge *et al.*, 2020). For our analyses, we used a previously imputed version of SLA data that was already carefully tested in terms of robustness in defining the trait dimensions underlying the abiotic stress tolerance strategies in the STS (Pavanetto *et al*., 2024*a*). SLA values for each hexagon were obtained from Pavanetto *et al*. (2024*b*), who calculated them using an approach similar to community-weighted means (Lavorel *et al*., 2008). In particular, SLA values were weighed based on the species relative abundances obtained from species occurrence data within each hexagon. Calculation of the weighed SLA values was performed separately for each PFT due to their distinct geographical distributions across the Northern Hemisphere and their different trait adaptations associated with given abiotic stress tolerance strategies in the STS (Pavanetto *et al*., 2024*a*). Finally, to ensure the most reliable calculation of the weighted mean of SLA values, hexagons that included fewer than ten total occurrences across all species per PFT were excluded from calculations. The final SLA data were available for all the species and assemblages in our dataset.

### Data analysis

Given that insect feeding guilds information in our dataset was aggregated at the species and at the assemblage level, hereafter we refer to feeding guilds in terms of ‘prevalence’, reflecting the mean compositional proportion of feeding guilds either at the species or at the assemblage level. We modelled the relative prevalence of insect feeding guilds (chewers, borers, miners) as a function of abiotic stress tolerance and SLA using Dirichlet regression (an extension of beta regression), which appropriately handles compositional response variables (Douma and Weedon, 2019). For each PFT (deciduous angiosperms, evergreen angiosperms, evergreen gymnosperms), we fitted separate models for the two stress tolerance axes. Each model included the stress tolerance axis score (either the waterlogging/cold–drought trade-off or the shade tolerance spectrum), scaled SLA, and their interaction as predictors and the Dirichlet-distributed vector of guild proportions as multivariate response. The considered subsets were (1) at the species level: deciduous angiosperms (*n* = 436), evergreen angiosperms (*n* = 101) and evergreen gymnosperms (*n* = 91); and (2) at the assemblage level: deciduous angiosperms (*n* = 2696), evergreen angiosperms (*n* = 1855) and evergreen gymnosperms (*n* = 2183). We used the common parameterization of Dirichlet regression, which models the log concentration parameters as linear functions of predictors. Models were fitted using the DirichletReg package in R (Maier, 2014).

To characterize how the effect of stress tolerance on guild composition varies with SLA, both at the species and at the assemblage level, we computed conditional effects at three SLA quantiles within each PFT: the 25th percentile (q25, representing low-SLA assemblages), the 50th percentile (q50, median) and the 75th percentile (q75, high-SLA assemblages). For each quantile, we computed the predicted change in guild prevalence (effect size, Δμ, in percentage points) across the observed range of the stress tolerance axis while holding SLA constant at the specified quantile value. This approach allows us to assess whether stress tolerance effects are modulated by the SLA context of the assemblage.

Finally, for the assemblage-level analysis, we assessed spatial autocorrelation in model residuals using Moran’s *I* statistic from each Dirichlet regression (Supplementary Data Note S1; Table S1). We found strong positive spatial autocorrelation (mean *I* = 0.66 across all models, Table S1), indicating that nearby assemblages exhibited more similar residuals than expected under spatial independence, suggesting that standard errors from the Dirichlet regressions were underestimated. To obtain valid confidence intervals that account for spatial non-independence, we implemented a spatial block bootstrap procedure. We partitioned the study area into approximately 500-km-diameter spatial blocks using k-means clustering on grid cell coordinates. For each of 500 bootstrap iterations, we resampled blocks with replacement, reconstructed the dataset from the selected blocks, refitted the Dirichlet regression models and computed conditional effects at each SLA quantile. Bootstrap 95 % confidence intervals were calculated from the 2.5th and 97.5th percentiles of the bootstrap distribution of effect estimates. Effects were considered statistically robust if their bootstrap confidence interval excluded zero. This approach preserves the spatial correlation structure within blocks while allowing inference regarding population parameters. As expected under a strong spatial autocorrelation signal, bootstrap standard errors averaged 10.2 percentage points compared to original (spatially unconstrained) standard errors of approximately 1–3 percentage points.

## RESULTS

**Fig. 1.**
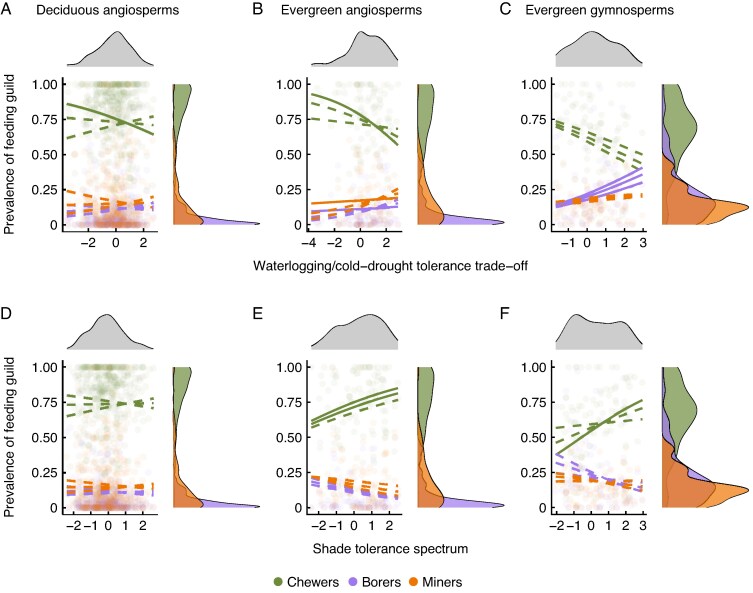
Prevalence of insect feeding guilds associated with woody plant species along the waterlogging/cold–drought tolerance trade-off (A–C) and the shade tolerance spectrum (D–F) for deciduous angiosperms (*n* = 436), evergreen angiosperms (*n* = 101) and evergreen gymnosperms (*n* = 91). Lines represent fitted values from Dirichlet regression for each insect feeding guild (chewers, borers, miners), with separate lines for SLA quantiles (q25, q50, q75) where the SLA × stress tolerance interaction was significant. Solid lines indicate significant relationships (at *P* ≤ 0.05); dashed lines indicate non-significant effects. Effect sizes are summarized in Supplementary Data Note S2 and Table S2.

At the plant species level, associations between stress tolerance and feeding guild prevalence were overall weak across all PFTs (Fig. 1). The interaction term included in the model was statistically significant for all six PFT × axis combinations (all *P* < 0.05; Supplementary Data Note S2, Table S2). However, predicted guild proportions along the stress axes at the 25th, 50th and 75th percentiles of SLA showed generally low effect sizes (Fig. 1; Table S2). Even in cases where significant effects occurred, effect sizes were typically small (Table S2). A detailed account of the effect sizes detected at the species level by PFT is presented in Note S2 and Table S2.

**Fig. 2.**
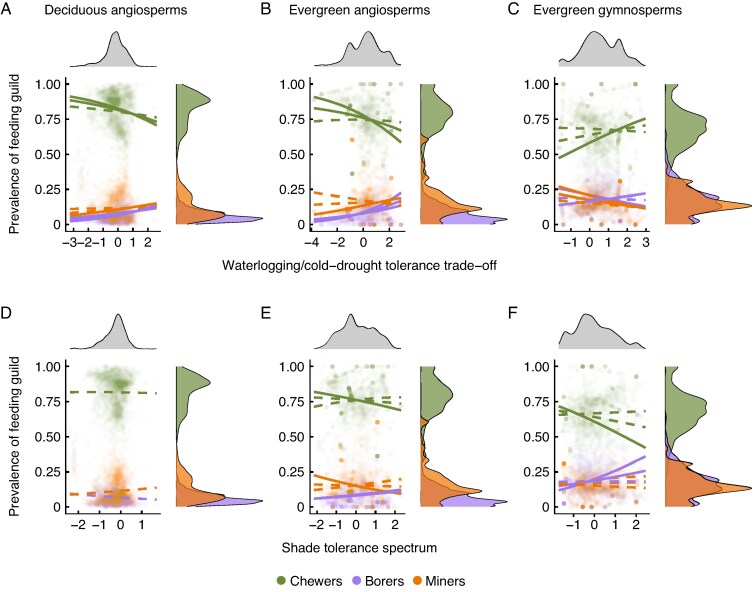
Prevalence of insect feeding guilds associated with woody plant assemblages along the waterlogging/cold–drought tolerance trade-off (A–C) and the shade tolerance spectrum (D–F) for deciduous angiosperms (*n* = 2696), evergreen angiosperms (*n* = 1885) and evergreen gymnosperms (*n* = 2183). Lines represent fitted values from Dirichlet regression for each insect feeding guild (chewers, borers, miners), with separate lines for SLA quantiles (q25, q50, q75) where the SLA × stress tolerance interaction was significant, or a single line at mean SLA for additive models (panel D only). Solid lines indicate effects that survived spatial block bootstrap correction (95 % CI excludes zero); dashed lines indicate non-significant effects. Statistical parameters are summarized in Supplementary Data Note S3 and Table S3.

We detected significant interactions between stress tolerance axes and SLA in predicting feeding guild composition across all PFTs and stress axes for assemblage-level data as well (Fig. 2; Supplementary Data Note S3, Table S3). The only exception was for deciduous angiosperm assemblages when including the shade tolerance spectrum as a predictor together with SLA (Fig. 2D). However, spatial block bootstrapping revealed that only 54.2 % (26 of 48) of the tested conditional effects were statistically robust after accounting for spatial autocorrelation (Fig. 2; Note S3, Table S3), compared to virtually 100 % significance detected in the spatial uncorrected analysis. A detailed account of the effect sizes detected in the assemblage-level analysis is presented in Note S3 and Table S3.

## DISCUSSION

We tested whether abiotic stress tolerance shapes insect herbivore guild composition, and whether SLA mediates this relationship. The answer is more complex than anticipated. We found a pervasive interaction between stress tolerance and SLA across PFTs (Figs 1 and 2), but closer scrutiny undermines simple mechanistic interpretation. We also find that herbivore communities are so strongly spatially structured that most apparent associations dissolve once this structure is accounted for (Supplementary Data Tables S1 and S3). Together, these results challenge the intuition that leaf structural traits straightforwardly link woody plants’ broad adaptive syndromes to tolerate abiotic stress to the prevalence of insect feeding guilds. In addition, only at the assemblage level did SLA quantiles produce visibly divergent predictions (Fig. 2). While the larger sample size of the assemblage-level analysis might have had some effect, this pattern is consistent with the general tendency for community-weighted metrics to amplify weak species-level signals into detectable community-level patterns (Pavanetto *et al*., 2024*b*; Westoby, 2025), suggesting that the SLA × stress tolerance interaction detected here operates primarily through assemblage composition. For this reason, below we discuss only the results relative to the assemblage-level analysis.

We found that the stress axis × SLA interaction was almost always significant across PFTs. However, the idiosyncratic patterns of how the prevalence of feeding guilds vary along stress axes at different SLA quantiles suggest that there are effects of abiotic stress tolerance on feeding guild prevalence that are not explained by SLA. For example, one of the largest robust effects detected (a 32 percentage point decline in chewer prevalence toward drought-tolerant evergreen angiosperm assemblages, Supplementary Data Table S3) occurs specifically among assemblages with low SLA values (q25). At first glance, this seems to support the hypothesis that structural leaf reinforcement deters chewing herbivores: drought-tolerant evergreen angiosperms invest in tough, low-SLA leaves with high fibre and sclerenchyma content (Wright *et al*., 2004; Poorter *et al*., 2009), and prevalence of chewers decreases in favour of other guilds. These might include borers and miners (drawing from our analysis), but also the prevalence of other guilds not included in our dataset can increase under specific stress imprints. For example, gallers tend to become more prevalent in habitats where drought is the main abiotic stress (Fernandes and Price, 1988). However, the conditional effect at low SLA compares assemblages that all have similarly low SLA values. If structural reinforcement were the mechanism, we would not need to condition on SLA; that is, low SLA would predict low chewer prevalence regardless of stress tolerance. Instead, what we observe is that drought tolerance affects guild composition through some pathways that SLA does not capture, and this effect is most pronounced when SLA is already low. One possibility is that plants deploy multiple defence strategies (e.g. chemical compounds, altered phenology) that covary with SLA. In this view, SLA values correspond to different contexts of baseline defence within which additional mechanisms operate. For instance, the costs for a plant to deploy low-SLA leaves might be compensated for by chemical defence through less costly compounds (Smilanich *et al*., 2016). Our interpretation also aligns with Liu *et al*. (2024), who found that herbivory intensity on woody plants possibly results through mechanisms that single traits fail to capture. SLA modulates the strength of stress tolerance effects on insect feeding guild prevalence, but it is not necessarily interposed between them.

Our findings also show strong spatial autocorrelation in model residuals (mean Moran’s *I* = 0.66, Supplementary Data Table S1) even after accounting for stress tolerance, SLA and their interaction. Assemblages sorted along the waterlogging/cold–drought trade-off and shade tolerance spectrum, as well as SLA, all show well-defined geographical patterns across the Northern Hemisphere (Pavanetto *et al*., 2024*b*), yet they fail to capture the geographical distribution of insect feeding guilds. This occurs even though herbivory intensity by ectothermic consumers, such as lepidopteran and hymenopteran larvae in our study, shows its own geographical patterns (Zvereva and Kozlov, 2021). This suggests that the distribution of insect feeding guilds depends on spatial processes (climate effects on herbivore physiology, historical biogeography, regional species pool assembly; Leather, 1986; Lewinsohn *et al*., 2005) that operate independently of the factors sorting assemblage-level plant adaptive strategies in geographical space.

We argue that the spatial autocorrelation detected in our study mostly reflects the biogeography of plant–herbivore associations. Herbivorous insects have limited dispersal, are constrained by host specificity, and are drawn from regional species pools shaped by evolutionary history, climate fluctuations and dispersal barriers unrelated to contemporary vegetation (Novotny *et al*., 2006; Forister *et al*., 2015). Chen *et al*. (2025), comparing plant–leaf miner networks across Europe and North America, found strong phylogenetic signals on both continents: closely related plants host similar leaf miner communities. This phylogenetic structuring means that spatial patterns in herbivore community composition partly mirror patterns in host plant phylogenetic composition (Novotny *et al*., 2010), an effect that our assemblage-level analysis cannot capture. Neighbouring assemblages share not just similar stress tolerance strategies and SLA values, but similar host plant lineages, and therefore similar herbivore faunas. Local trait-based filtering thus operates against a backdrop of regional species pool constraints: a plant community with traits and stress imprint that should favour specific insect feeding guilds will not exhibit this pattern if the species belonging to those guilds, or hosts, are not available in the regional species pool. Robinson et al. (2023) found that what varies most systematically across macroecological gradients is not the mean intensity of plant–herbivore interactions but the composition of regional herbivore faunas, consistent with our finding that spatial structure dominates over stress and trait effects.

## LIMITATIONS

Our reliance on SLA as the focal trait is limiting. On the one hand, SLA data are readily available for many species worldwide, justifying the use of this trait in broad comparative analyses such as the one presented here. On the other hand, SLA integrates multiple leaf properties and covaries with chemical defences, leaf lifespan and palatability (Wright *et al*., 2004) in ways our analysis cannot disentangle (see Smilanich *et al*., 2016). Direct measurements of secondary chemistry and nutritional quality would help isolate specific defensive mechanisms (Carmona *et al*., 2011). Similarly, our guild classifications collapse substantial biological diversity; different herbivore species within a guild may respond differently to plant traits. The patterns observed varied substantially among PFTs. Evergreen angiosperms showed the strongest SLA × stress tolerance interactions, probably because they span the widest range of leaf economics – from tender leaves in moister habitats to leathery Mediterranean sclerophylls (Poorter *et al*., 2009). Deciduous angiosperms, constrained to a fast-return strategy, lack the low-SLA extreme and showed weaker effects. For evergreen gymnosperms we cannot exclude a predominant role of chemical rather than structural defences (e.g. oleoresins, tannins, terpenes) that may override the influence of SLA (Franceschi *et al*., 2005; Keeling and Bohlmann, 2006). These PFT-specific patterns suggest that the SLA × stress tolerance interaction is contingent on the underlying leaf economic strategy and phylogenetic constraints of each functional group. We also recognize other potential limitations. First, temporal dynamics and ontogeny influence both plant traits and herbivory intensity (Coley, 1980; Barton *et al*., 2019; Wetzel *et al*., 2023). Second, some insects exhibit flexible feeding behaviours – switching among tissues or guilds across life stages (Connor and Taverner, 1997; Powell *et al*., 1998) – which may blur guild boundaries that in turn might be too broadly defined (such as in the case of borers in our classification, Table 1). Third, top-down processes can modulate herbivore impacts in non-additive ways (Moreira *et al*., 2012, 2013; Fernández de Bobadilla *et al*., 2021; Karssemeijer *et al*., 2022; Wise, 2023).

## CONCLUSIONS

Abiotic stress tolerance and SLA interact to shape herbivore guild composition, but the mechanism is not what the leaf economics framework would predict. SLA does not mediate the stress tolerance–herbivore relationship; rather, it defines a context within which other unidentified factors operate. Meanwhile, herbivore communities are so strongly spatially structured that most apparent trait–composition associations are not robust to correction for spatial non-independence. This spatial structure probably carries ecological information about dispersal limitations, host specificity and the regional assembly of herbivore faunas. Understanding plant–herbivore interactions at macroecological scales will require moving beyond single-trait predictors toward multivariate defence strategies and integrating local trait-based filtering with biogeographical processes.

## Supplementary Material

mcag065_Supplementary_Data

## References

[mcag065-B1] Agrawal AA, Fishbein M. 2006. Plant defense syndromes. Ecology 87: S132–S149. doi:10.1890/0012-9658(2006)87[132:PDS]2.0.CO;2 16922309

[mcag065-B2] Agrawal AA, Maron JL. 2022. Long-term impacts of insect herbivores on plant populations and communities. Journal of Ecology 110: 2800–2811. doi:10.1111/1365-2745.13996

[mcag065-B3] Barton KE, Edwards KF, Koricheva J. 2019. Shifts in woody plant defence syndromes during leaf development. Functional Ecology 33: 2095–2104. doi:10.1111/1365-2435.13435

[mcag065-B4] Barton KE, Hanley ME. 2013. Seedling–herbivore interactions: insights into plant defence and regeneration patterns. Annals of Botany 112: 643–650. doi:10.1093/aob/mct139 23925939 PMC3736773

[mcag065-B5] Bernays EA . 1998. Evolution of feeding behavior in insect herbivores: success seen as different ways to eat without being eaten. BioScience 48: 35–44. doi:10.2307/1313226

[mcag065-B6] Boivin T, Doublet V, Candau J-N. 2019. The ecology of predispersal insect herbivory on tree reproductive structures in natural forest ecosystems. Insect Science 26: 182–198. doi:10.1111/1744-7917.12549 29082661

[mcag065-B7] Brown JW . 2018. Patterns of Lepidoptera herbivory on conifers in the New World. Journal of Asia-Pacific Biodiversity 11: 1–10. doi:10.1016/j.japb.2018.01.008

[mcag065-B8] Carmona CP, Bueno CG, Toussaint A, et al 2021. Fine-root traits in the global spectrum of plant form and function. Nature 597: 683–687. doi:10.1038/s41586-021-03871-y 34588667

[mcag065-B9] Carmona D, Lajeunesse MJ, Johnson MTJ. 2011. Plant traits that predict resistance to herbivores. Functional Ecology 25: 358–367. doi:10.1111/j.1365-2435.2010.01794.x

[mcag065-B10] Cerdeira-Pérez A, Laanisto L, Puglielli G. 2025. InsectGUILD: feeding guilds of lepidopteran and hymenopteran larvae consuming Northern Hemisphere woody plants. Scientific Data 12: 887. doi:10.1038/s41597-025-05229-9 40436885 PMC12119911

[mcag065-B11] Chen X, Dai X, Guo Q, Eiseman CS. 2025. Convergence of plant-leafminer associations on two continents. Global Ecology and Conservation 61: e03656. doi:10.1016/j.gecco.2025.e03656

[mcag065-B12] Coley PD . 1980. Effects of leaf age and plant life history patterns on herbivory. Nature 284: 545–546. doi:10.1038/284545a0

[mcag065-B13] Connor EF, Taverner MP. 1997. The evolution and adaptive significance of the leaf-mining habit. Oikos 79: 6–25. doi:10.2307/3546085

[mcag065-B14] Douma JC, Weedon JT. 2019. Analysing continuous proportions in ecology and evolution: a practical introduction to beta and Dirichlet regression. Methods in Ecology and Evolution 10: 1412–1430. doi:10.1111/2041-210X.13234

[mcag065-B15] Fernandes GW, Price PW. 1988. Biogeographical gradients in galling species richness. Oecologia 76: 161–167. doi:10.1007/BF00379948 28312192

[mcag065-B16] Fernández de Bobadilla M, Bourne ME, Bloem J, et al 2021. Insect species richness affects plant responses to multi-herbivore attack. New Phytologist 231: 2333–2345. doi:10.1111/nph.17228 33484613 PMC8451852

[mcag065-B17] Forister ML, Novotny V, Panorska AK, et al 2015. The global distribution of diet breadth in insect herbivores. Proceedings of the National Academy of Sciences of the United States of America 112: 442–447. doi:10.1073/pnas.1423042112 25548168 PMC4299246

[mcag065-B18] Franceschi VR, Krokene P, Christiansen E, Krekling T. 2005. Anatomical and chemical defenses of conifer bark against bark beetles and other pests. New Phytologist 167: 353–376. doi:10.1111/j.1469-8137.2005.01436.x 15998390

[mcag065-B19] Garcia A, Martinez M, Diaz I, Santamaria ME. 2021. The price of the induced defense against pests: a meta-analysis. Frontiers in Plant Science 11: 615122. doi:10.3389/fpls.2020.615122 33552106 PMC7859116

[mcag065-B20] Haavik LJ, Stephen FM. 2023. Insect ecology. In: Entomology. Forest entomology and pathology. Berlin: Springer International Publishing, 91–113.

[mcag065-B21] Karssemeijer PN, Winzen L, van Loon JJA, Dicke M. 2022. Leaf-chewing herbivores affect preference and performance of a specialist root herbivore. Oecologia 199: 243–255. doi:10.1007/s00442-022-05132-9 35192063 PMC9226102

[mcag065-B22] Kattge J, Bönisch G, Díaz S et al 2020. TRY plant trait database – enhanced coverage and open access. Global Change Biology 26: 119–188. doi:10.1111/gcb.14904 31891233

[mcag065-B23] Keeling CI, Bohlmann J. 2006. Diterpene resin acids in conifers. Phytochemistry 67: 2415–2423. doi:10.1016/j.phytochem.2006.08.019 16996548

[mcag065-B24] Koricheva J . 2002. Meta-analysis of sources of variation in fitness costs of plant antiherbivore defenses. Ecology 83: 176–190. doi:10.1890/0012-9658(2002)083[0176:MAOSOV]2.0.CO;2

[mcag065-B25] Koricheva J, Larsson S, Haukioja E. 1998. Insect performance on experimentally stressed woody plants: a meta-analysis. Annual Review of Entomology 43: 195–216. doi:10.1146/annurev.ento.43.1.195 15012389

[mcag065-B26] Laanisto L, Niinemets Ü. 2015. Polytolerance to abiotic stresses: how universal is the shade–drought tolerance trade-off in woody species? Global Ecology and Biogeography 24: 571–580. doi:10.1111/geb.12288 29367836 PMC5777592

[mcag065-B27] Lavorel S, Grigulis K, McIntyre S, et al 2008. Assessing functional diversity in the field—methodology matters!. Functional Ecology 22: 134–147. doi:10.1111/j.1365-2435.2007.01339.x

[mcag065-B28] Leather SR . 1986. Insect species richness of the British Rosaceae: the importance of host range, plant architecture, age of establishment, taxonomic isolation and species-area relationships. Journal of Animal Ecology 55: 841–860. doi:10.2307/4420

[mcag065-B29] Lewinsohn TM, Novotny V, Basset Y. 2005. Insects on plants: diversity of herbivore assemblages revisited. Annual Review of Ecology, Evolution, and Systematics 36: 597–620. doi:10.1146/annurev.ecolsys.36.091704.175520

[mcag065-B30] Liu M, Jiang P, Chase JM, Liu X. 2024. Global insect herbivory and its response to climate change. Current Biology 34: 2558–2569.e3. doi:10.1016/j.cub.2024.04.062 38776900

[mcag065-B31] Maier JM . 2014. *DirichletReg: Dirichlet regression for compositional data in R*. Research Report Series / Department of Statistics and Mathematics, no. 125. doi:10.57938/ad3142d3-2fcd-4c37-aec6-8e0bd7d077e1.

[mcag065-B32] Marquis RJ . 1992. The selective impact of herbivores. In: Plant resistance to herbivores and pathogens: ecology, evolution, and genetics. Chicago, IL: University of Chicago Press, 301–325.

[mcag065-B33] Moreira X, Lundborg L, Zas R, Carrillo-Gavilán A, Borg-Karlson A-K, Sampedro L. 2013. Inducibility of chemical defences by two chewing insect herbivores in pine trees is specific to targeted plant tissue, particular herbivore and defensive trait. Phytochemistry 94: 113–122. doi:10.1016/j.phytochem.2013.05.008 23768645

[mcag065-B34] Moreira X, Mooney KA, Zas R, Sampedro L. 2012. Bottom-up effects of host-plant species diversity and top-down effects of ants interactively increase plant performance. Proceedings of the Royal Society of London: Series B, Biological Sciences 279: 4464–4472. doi:10.1098/rspb.2012.0893 PMC3479792 22951745

[mcag065-B35] Niinemets Ü . 2010. Responses of forest trees to single and multiple environmental stresses from seedlings to mature plants: past stress history, stress interactions, tolerance and acclimation. Forest Ecology and Management 260: 1623–1639. doi:10.1016/j.foreco.2010.07.054

[mcag065-B36] Niinemets Ü, Valladares F. 2006. Tolerance to shade, drought, and waterlogging of temperate northern hemisphere trees and shrubs. Ecological Monographs 76: 521–547. doi:10.1890/0012-9615(2006)076[0521:TTSDAW]2.0.CO;2

[mcag065-B37] Novotny V, Drozd P, Miller SE, et al 2006. Why are there so many species of herbivorous insects in tropical rainforests? Science 313: 1115–1118. doi:10.1126/science.1129237 16840659

[mcag065-B38] Novotny V, Miller SE, Baje L, et al 2010. Guild-specific patterns of species richness and host specialization in plant–herbivore food webs from a tropical forest. Journal of Animal Ecology 79: 1193–1203. doi:10.1111/j.1365-2656.2010.01728.x 20673235

[mcag065-B39] Pavanetto N, Carmona CP, Laanisto L, Niinemets Ü, Puglielli G. 2024a. Trait dimensions of abiotic stress tolerance in woody plants of the Northern Hemisphere. Global Ecology and Biogeography 33: 272–285. doi:10.1111/geb.13788

[mcag065-B40] Pavanetto N, Niinemets Ü, Rueda M, Puglielli G. 2024b. Macroecology of abiotic stress tolerance in woody plants of the northern hemisphere: tolerance biomes and polytolerance hotspots. Ecology Letters 27: e70016. doi:10.1111/ele.70016 39623739 PMC11612541

[mcag065-B41] Peeters PJ . 2002. Correlations between leaf structural traits and the densities of herbivorous insect guilds. Biological Journal of the Linnean Society 77: 43–65. doi:10.1046/j.1095-8312.2002.00091.x

[mcag065-B42] Peeters PJ, Sanson G, Read J. 2007. Leaf biomechanical properties and the densities of herbivorous insect guilds. Functional Ecology 21: 246–255. doi:10.1111/j.1365-2435.2006.01223.x

[mcag065-B43] Poorter H, Niinemets Ü, Poorter L, Wright IJ, Villar R. 2009. Causes and consequences of variation in leaf mass per area (LMA): a meta-analysis. New Phytologist 182: 565–588. doi:10.1111/j.1469-8137.2009.02830.x 19434804

[mcag065-B44] Powell J, Mitter C, Farrell B. 1998. Evolution of larval food preferences in Lepidoptera. eds. Handbook of zoology. Lepidoptera. Moths and butterflies, 403–422.

[mcag065-B45] Puglielli G, Hutchings MJ, Laanisto L. 2021. The triangular space of abiotic stress tolerance in woody species: a unified trade-off model. New Phytologist 229: 1354–1362. doi:10.1111/nph.16952 32989754

[mcag065-B46] Puglielli G, Pavanetto N, Laanisto L. 2022. Towards a “*periodic table*” of abiotic stress tolerance strategies of woody plants. Flora 292: 152089. doi:10.1016/j.flora.2022.152089

[mcag065-B47] Robinson ML, Hahn PG, Inouye BD et al 2023. Plant size, latitude, and phylogeny explain within-population variability in herbivory. Science 382: 679–683. doi:10.1126/science.adh8830 37943897

[mcag065-B48] Simberloff D, Dayan T. 1991. The guild concept and the structure of ecological communities. Annual Review of Ecology, Evolution, and Systematics 22: 115–143. doi:10.1146/annurev.es.22.110191.000555

[mcag065-B49] Sinclair RJ, Hughes L. 2010. Leaf miners: the hidden herbivores. Austral Ecology 35: 300–313. doi:10.1111/j.1442-9993.2009.02039.x

[mcag065-B50] Smilanich AM, Fincher RM, Dyer LA. 2016. Does plant apparency matter? Thirty years of data provide limited support but reveal clear patterns of the effects of plant chemistry on herbivores. New Phytologist 210: 1044–1057. doi:10.1111/nph.13875 26889654

[mcag065-B51] Stam JM, Kroes A, Li Y, et al 2014. Plant interactions with multiple insect herbivores: from community to genes. Annual Review of Plant Biology 65: 689–713. doi:10.1146/annurev-arplant-050213-035937 24313843

[mcag065-B52] Tooker JF, Giron D. 2020. The evolution of endophagy in herbivorous insects. Frontiers in Plant Science 11: 581816. doi:10.3389/fpls.2020.581816 33250909 PMC7673406

[mcag065-B53] Westoby M . 2025. Trait-based ecology, trait-free ecology, and in between. New Phytologist 245: 33–39. doi:10.1111/nph.20197 39410833

[mcag065-B54] Wetzel WC, Inouye BD, Hahn PG, Whitehead SR, Underwood N. 2023. Variability in plant–herbivore interactions. Annual Review of Ecology, Evolution, and Systematics 54: 451–474. doi:10.1146/annurev-ecolsys-102221-045015

[mcag065-B55] Wise MJ . 2023. Why fitness impacts of different herbivores may combine nonadditively, and why it matters to the ecology and evolution of plant-herbivore communities. Plant Ecology and Evolution 156: 13–28. doi:10.5091/plecevo.95982

[mcag065-B56] Wright IJ, Reich PB, Westoby M, et al 2004. The worldwide leaf economics spectrum. Nature 428: 821–827. doi:10.1038/nature02403 15103368

[mcag065-B57] Züst T, Agrawal AA. 2017. Trade-offs between plant growth and defense against insect herbivory: an emerging mechanistic synthesis. Annual Review of Plant Biology 68: 513–534. doi:10.1146/annurev-arplant-042916-040856 28142282

[mcag065-B58] Zvereva EL, Kozlov MV. 2021. Latitudinal gradient in the intensity of biotic interactions in terrestrial ecosystems: sources of variation and differences from the diversity gradient revealed by meta-analysis. Ecology Letters 24: 2506–2520. doi:10.1111/ele.13851 34322961

