# Supplementary material for: Specific leaf area modulates but does not explain the association between abiotic stress tolerance and insect feeding guild prevalence in Northern Hemisphere woody plants
Source: Ann Bot. 2026 Mar 19;137(7):2248–56. doi: 10.1093/aob/mcag065 (PMC13319342; doi:10.1093/aob/mcag065)
Supplement: mcag065_Supplementary_Data [file mcag065_supplementary_data.docx]

**Supporting information for the article:** Insect feeding guilds in relation to abiotic stress tolerance in Northern Hemisphere woody plants

**Authors:** Andrea Cerdeira-Pérez, Lauri Laanisto, Nicola Pavanetto, Giacomo Puglielli

**The following Supporting Information is available for this article:**

**Note S1.** Spatial autocorrelation assessment

**Table S1.** Moran's *I* statistics for spatial autocorrelation in Dirichlet regression residuals.

**Note S2.** Detailed species-level analysis effect sizes.

**Table S2.** Species-level effect sizes.

**Note S3.** Detailed assemblage-level analysis effect sizes with bootstrap correction.

**Table S3.** Assemblage-level effect sizes with bootstrap correction.

**Note S1. Spatial autocorrelation assessment**

We assessed spatial autocorrelation in Dirichlet regression residuals using Moran's *I* statistic. For each PFT × stress axis combination, we fitted the full interaction model (Y ~ STS × SLA) and extracted two types of residuals: (1) ILR-transformed residuals: Compositional data were transformed using the isometric log-ratio (ILR) transformation to remove the unit-sum constraint before computing residuals. For three-part compositions (chewers, borers, miners), this yields two unconstrained ILR components. (2) Response residuals: Raw differences between observed and fitted guild proportions, computed separately for each guild to aid interpretation. Spatial weights were constructed using *k*-nearest neighbours (*k* = 6) based on hexagonal grid cell centroids, with row-standardised weights. Moran's *I* was computed using the *spdep* package in R (Bivand & Wong, 2018), with inference based on the analytical expectation and variance under the normality assumption.

We found substantial positive spatial autocorrelation in all models (**Table S1**). Moran's *I* ranged from 0.51 to 0.84 across residual types and models, with a mean of 0.66. All tests were highly significant (*p* < 0.001), indicating that nearby assemblages exhibited more similar residuals than expected under spatial independence. Autocorrelation was highest for deciduous angiosperms (mean *I* = 0.73) and lowest for evergreen gymnosperms (mean *I* = 0.59).

**Table S1.** Moran's *I* statistics for spatial autocorrelation in Dirichlet regression residuals. Dirichlet regression models were fitted using either stress axis, specific leaf area and their interaction as predictors for the three considered plant functional types. ILR = isometric log-ratio transformed residuals; Response = raw guild-specific residuals. Bold Moran’s I values indicate significance at *p* < 0.001.

|  |  |  |  | **Deciduous angiosperms** | |  | **Evergreen angiosperms** | |  | **Evergreen gymnosperms** | |
| --- | --- | --- | --- | --- | --- | --- | --- | --- | --- | --- | --- |
| **Stress axis** | **Residual type** | **Variable** |  | Moran's I | z-score |  | Moran's I | z-score |  | Moran's I | z-score |
| **Waterlogging/cold-drought trade-off** | **ILR** | **ILR1** |  | **0.743** | 70.73 |  | **0.697** | 55.93 |  | **0.51** | 43.94 |
|  |  | **ILR2** |  | **0.537** | 51.12 |  | **0.679** | 54.46 |  | **0.674** | 57.98 |
|  | **Response** | **Chewers** |  | **0.829** | 78.91 |  | **0.653** | 52.38 |  | **0.553** | 47.59 |
|  |  | **Borers** |  | **0.688** | 65.69 |  | **0.627** | 50.34 |  | **0.554** | 47.68 |
|  |  | **Miners** |  | **0.829** | 78.87 |  | **0.627** | 50.36 |  | **0.599** | 51.54 |
| **Shade tolerance spectrum** | **ILR** | **ILR1** |  | **0.754** | 71.82 |  | **0.709** | 56.9 |  | **0.527** | 45.39 |
|  |  | **ILR2** |  | **0.521** | 49.64 |  | **0.675** | 54.19 |  | **0.669** | 57.59 |
|  | **Response** | **Chewers** |  | **0.84** | 79.96 |  | **0.689** | 55.27 |  | **0.583** | 50.18 |
|  |  | **Borers** |  | **0.704** | 67.18 |  | **0.658** | 52.88 |  | **0.557** | 47.94 |
|  |  | **Miners** |  | **0.83** | 78.96 |  | **0.654** | 52.52 |  | **0.651** | 55.94 |

**Note S2. Detailed account of effect sizes detected in the species-level analysis**

All the effect sizes are reported in **Table S2**. For deciduous angiosperms (n = 436 species), only chewer prevalence at high SLA showed a significant decline along the waterlogging/cold–drought axis (q75: Δμ = −21.6 pp, p < 0.05); no other effects were significant for this PFT. Evergreen angiosperms (n = 101 species) displayed scattered significant effects: chewer prevalence declined toward drought tolerance at high SLA (q75: Δμ = −36.4 pp), while borers and miners showed modest increases at low SLA (q25: Δμ = +3.3 pp and +4.0 pp, respectively). Along the shade tolerance spectrum, chewer prevalence increased at median and high SLA (q50: Δμ = +21.9 pp; q75: Δμ = +23.4 pp). Evergreen gymnosperms (n = 91 species) showed the most consistent species-level pattern: borer prevalence increased toward drought tolerance across all SLA quantiles (q25: Δμ = +17.5 pp; q50: Δμ = +22.3 pp; q75: Δμ = +26.5 pp), while chewer prevalence increased toward shade tolerance only at high SLA (q75: Δμ = +39.1 pp). The limited number of significant effects at the species level, combined with the absence of consistent patterns across PFTs, suggests that individual species traits are weak predictors of herbivore guild composition. This contrasts sharply with the assemblage-level results, where aggregating across co-occurring species revealed stronger and more consistent environment–herbivory relationships.

**Table S2.** Species-level effect sizes for insect feeding guild prevalence along abiotic stress tolerance axes for the considered plant functional types. Effect sizes (Δμ) from species-level Dirichlet regressions with SLA × stress tolerance interactions represent the change in guild prevalence (percentage points, pp) across the observed stress tolerance range at each SLA quantile (q). Significance was determined by Wald tests on model coefficients (*p* < 0.05). Sample sizes: deciduous angiosperms (DA), *n* = 436 species; evergreen angiosperms (EA), *n* = 101 species; evergreen gymnosperms (EG), *n* = 91 species. Bold values indicate significant effects.

|  |  |  |  | **Δμ (pp)** | | |
| --- | --- | --- | --- | --- | --- | --- |
| **Stress axis** | **Guild** | **SLA level** |  | **Deciduous angiosperms** | **Evergreen angiosperms** | **Evergreen gymnosperms** |
| **Waterlogging/cold-drought trade-off** | **Chewers** | **q25** |  | 15.4 | −7.3 | −23.8 |
|  |  | **q50** |  | −5.0 | −24.1 | −28.3 |
|  |  | **q75** |  | **−21.6** | **−36.4** | −31.9 |
|  | **Borers** | **q25** |  | −3.7 | **3.3** | **17.5** |
|  |  | **q50** |  | 3.4 | 9.8 | **22.3** |
|  |  | **q75** |  | 9.6 | 14.8 | **26.5** |
|  | **Miners** | **q25** |  | −11.7 | **4** | 6.3 |
|  |  | **q50** |  | 1.7 | 14.3 | 6 |
|  |  | **q75** |  | 12 | 21.6 | 5.5 |
|  |  |  |  |  |  |  |
| **Shade tolerance spectrum** | **Chewers** | **q25** |  | 12.6 | 19.7 | 6.2 |
|  |  | **q50** |  | 1.1 | **21.9** | 24.7 |
|  |  | **q75** |  | −9.1 | **23.4** | **39.1** |
|  | **Borers** | **q25** |  | −6.5 | −13.3 | −6.8 |
|  |  | **q50** |  | −1.7 | −11.4 | −17.8 |
|  |  | **q75** |  | 2.6 | −9.9 | −26.9 |
|  | **Miners** | **q25** |  | −6.1 | −6.4 | 0.6 |
|  |  | **q50** |  | 0.6 | −10.5 | −6.8 |
|  |  | **q75** |  | 6.5 | −13.5 | −12.2 |

**Note S3. Assemblage-level effect sizes with bootstrap correction**

All the effect sizes and their bootstrap corrections are reported in **Table S3**. Deciduous angiosperms (n = 2,696 assemblages) showed consistent shifts in guild composition along the waterlogging/cold–drought trade-off, with the interaction term significant (LR test p < 0.05). Chewer prevalence declined toward drought-tolerant assemblages, with the effect strengthening at higher SLA quantiles: the decline was not robust at low SLA (q25: Δμ = −7.8 pp, 95% CI: −17.8 to +1.0), but was robust at median SLA (q50: Δμ = −15.2 pp, 95% CI: −24.5 to −5.8) and high SLA (q75: Δμ = −20.4 pp, 95% CI: −30.7 to −10.1). Borer prevalence increased toward drought tolerance, with robust effects across all SLA quantiles (q25: Δμ = +7.0 pp, 95% CI: +2.4 to +11.6; q50: Δμ = +9.8 pp, 95% CI: +5.7 to +13.7; q75: Δμ = +11.7 pp, 95% CI: +7.0 to +15.6). Miner prevalence increased significantly only at high SLA (q75: Δμ = +8.7 pp, 95% CI: +1.5 to +16.4), with non-significant trends at lower quantiles. Along the shade tolerance spectrum, the interaction term was not significant, and an additive model was fitted. No guild showed robust effects along this axis at mean SLA (chewers: Δμ = −0.8 pp, 95% CI: −12.5 to +10.5; borers: Δμ = −4.1 pp, 95% CI: −9.2 to +0.7; miners: Δμ = +4.9 pp, 95% CI: −1.6 to +12.1).

Evergreen angiosperms (*n* = 1,885 assemblages) displayed the largest effect sizes and strongest evidence for SLA-dependent relationships. Along the waterlogging/cold–drought trade-off, chewer prevalence declined sharply, with the effect strongest at low SLA (q25: Δμ = −32.2 pp, 95% CI: −43.0 to −24.0) representing the largest robust effect detected in this study. The decline remained robust at median SLA (q50: Δμ = −15.7 pp, 95% CI: −28.1 to −7.3) but was not significant at high SLA (q75: Δμ = −0.7 pp, 95% CI: −18.1 to +13.6). Borer prevalence increased toward drought tolerance with robust effects across all SLA quantiles (q25: Δμ = +20.5 pp, 95% CI: +13.4 to +29.4; q50: Δμ = +14.1 pp, 95% CI: +9.1 to +20.5; q75: Δμ = +9.8 pp, 95% CI: +6.2 to +14.3). Miner prevalence increased significantly at low SLA (q25: Δμ = +11.8 pp, 95% CI: +6.3 to +18.7) but showed non-significant or opposing trends at higher quantiles. Along the shade tolerance spectrum, several effects were robust despite smaller magnitudes. Chewer prevalence declined significantly at high SLA (q75: Δμ = −13.1 pp, 95% CI: −24.9 to −0.2). Borer prevalence increased across all SLA quantiles (q25: Δμ = +6.4 pp, 95% CI: +0.1 to +15.2; q50: Δμ = +5.9 pp, 95% CI: +1.1 to +11.9; q75: Δμ = +5.4 pp, 95% CI: +0.9 to +10.2). Miner prevalence decreased at low SLA (q25: Δμ = −13.3 pp, 95% CI: −21.1 to −1.2).

Evergreen gymnosperms (n = 2,183 assemblages) presented contrasting patterns between stress axes. Along the waterlogging/cold–drought trade-off, guild composition showed strong but opposing responses at different SLA quantiles. Chewer prevalence increased toward drought tolerance at low SLA (q25: Δμ = +28.2 pp, 95% CI: +11.0 to +43.7) but showed no significant change at median or high SLA. Borer prevalence showed a reversal across SLA quantiles: declining at low SLA (q25: Δμ = −12.6 pp, 95% CI: −21.0 to −3.3) but increasing at high SLA (q75: Δμ = +8.2 pp, 95% CI: +1.0 to +20.7). Miner prevalence declined consistently, with robust effects at low and median SLA (q25: Δμ = −15.6 pp, 95% CI: −23.2 to −7.2; q50: Δμ = −9.8 pp, 95% CI: −17.0 to −0.7). Along the shade tolerance spectrum, chewer prevalence declined sharply at low SLA (q25: Δμ = −29.6 pp, 95% CI: −42.9 to −10.8), while borer prevalence increased (q25: Δμ = +24.1 pp, 95% CI: +11.3 to +34.0; q50: Δμ = +10.8 pp, 95% CI: +0.7 to +20.2). Miner prevalence showed no robust effects along this axis.

**Table S3.** Effect sizes from assemblage-level Dirichlet regressions with spatial block bootstrap correction. Effect sizes (Δμ) represent the change in guild prevalence (percentage points, pp) across the observed stress tolerance range. For models where the SLA × stress tolerance interaction was significant, effects are reported at three SLA quantiles (q25, q50, q75). For deciduous angiosperms assemblages along the shade tolerance spectrum, the interaction was not significant, so an additive model was used, and effects are reported at mean SLA only. Sample sizes: deciduous angiosperms, n = 2,696 assemblages; evergreen angiosperms, n = 1,885 assemblages; evergreen gymnosperms, n = 2,183 assemblages. Bold values indicate significant effects after bootstrap correction (95% CI excluded zero).

|  |  |  |  | **Deciduous angiosperms** | |  | **Evergreen angiosperms** | |  | **Evergreen gymnosperms** | |
| --- | --- | --- | --- | --- | --- | --- | --- | --- | --- | --- | --- |
| **Axis** | **Guild** | **SLA level** |  | **Δμ** | **95% CI** |  | **Δμ** | **95% CI** |  | **Δμ** | **95% CI** |
| **Waterlogging/cold-drought trade-off** | **Chewers** | **q25** |  | −7.8 | −17.8 to +1.0 |  | **−32.2** | **−43.0 to −24.0** |  | **28.2** | **+11.0 to +43.7** |
|  |  | **q50** |  | **−15.2** | **−24.5 to −5.8** |  | **−15.7** | **−28.1 to −7.3** |  | 11.1 | −8.1 to +25.7 |
|  |  | **q75** |  | **−20.4** | **−30.7 to −10.1** |  | −0.7 | −18.1 to +13.6 |  | −3.1 | −24.9 to +11.2 |
|  | **Borers** | **q25** |  | **7** | **+2.4 to +11.6** |  | **20.5** | **+13.4 to +29.4** |  | **−12.6** | **−21.0 to −3.3** |
|  |  | **q50** |  | **9.8** | **+5.7 to +13.7** |  | **14.1** | **+9.1 to +20.5** |  | −1.4 | −8.8 to +9.1 |
|  |  | **q75** |  | **11.7** | **+7.0 to +15.6** |  | **9.8** | **+6.2 to +14.3** |  | **8.2** | **+1.0 to +20.7** |
|  | **Miners** | **q25** |  | 0.8 | −4.7 to +6.7 |  | **11.8** | **+6.3 to +18.7** |  | **−15.6** | **−23.2 to −7.2** |
|  |  | **q50** |  | 5.4 | −0.6 to +11.8 |  | 1.6 | −4.4 to +9.3 |  | **−9.8** | **−17.0 to −0.7** |
|  |  | **q75** |  | **8.7** | **+1.5 to +16.4** |  | −9.2 | −21.1 to +4.5 |  | −5.1 | −12.2 to +4.7 |
|  |  |  |  |  |  |  |  |  |  |  |  |
| **Shade tolerance spectrum** | **Chewers** | **q25** |  | −0.8 | −12.5 to +10.5 |  | 6.9 | −13.5 to +20.5 |  | **−29.6** | **−42.9 to −10.8** |
|  |  | **q50** |  |  |  |  | −4.4 | −17.9 to +6.5 |  | −11.8 | −24.6 to +4.5 |
|  |  | **q75** |  |  |  |  | **−13.1** | **−24.9 to −0.2** |  | 2.6 | −8.9 to +16.1 |
|  | **Borers** | **q25** |  |  |  |  | **6.4** | **+0.1 to +15.2** |  | **24.1** | **+11.3 to +34.0** |
|  |  | **q50** |  | −4.1 | −9.2 to +0.7 |  | **5.9** | **+1.1 to +11.9** |  | **10.8** | **+0.7 to +20.2** |
|  |  | **q75** |  |  |  |  | **5.4** | **+0.9 to +10.2** |  | 0.3 | −8.0 to +8.3 |
|  | **Miners** | **q25** |  |  |  |  | **−13.3** | **−21.1 to −1.2** |  | 5.5 | −2.8 to +13.8 |
|  |  | **q50** |  |  |  |  | −1.5 | −8.1 to +6.5 |  | 1 | −6.2 to +7.4 |
|  |  | **q75** |  | 4.9 | −1.6 to +12.1 |  | 7.7 | −2.1 to +16.7 |  | −2.9 | −8.9 to +2.2 |

# References

# Bivand, R. & Wong, D.W.S. (2018) Comparing implementations of global and local indicators of spatial association. TEST, 27, 716–748.
